# Peritonitis bacteriana espontánea en ascitis cardiaca: Presentación de un caso y revisión de la literatura

**DOI:** 10.23938/ASSN.1153

**Published:** 2026-02-17

**Authors:** Virginia González Hidalgo, Rocío Sofía González Gallego, Ruth María Cicuéndez Trilla, Shaila Alonso Rodríguez

**Affiliations:** Servicio de Medicina Interna Hospital General de Fuerteventura Virgen de la Peña Servicio Canario de la Salud Puerto del Rosario, Fuerteventura España

**Keywords:** Ascitis, Peritonitis bacteriana espontánea, Proteínas, Insuficiencia cardiaca, Ascites, Spontaneous bacterial peritonitis, Proteins, Heart failure

## Abstract

La ascitis puede tener origen cardiaco en un 5% de los casos. Aunque el elevado contenido proteico del líquido ascítico previene la peritonitis bacteriana espontánea en ascitis cardiaca, esta puede presentarse en escenarios de insuficiencia cardíaca grave con congestión e hipoperfusión intestinal.

Presentamos el caso de un varón de 65 años con insuficiencia cardíaca avanzada, fracción de eyección del 19%, insuficiencia tricuspidea grave e hipertensión pulmonar, que consultó por disnea y aumento del perímetro abdominal. La ecografía descartó hipertensión portal y cirrosis. El análisis del líquido ascítico (proteínas totales 3,5 g/dL, gradiente de albúmina sérica-ascitis 2,2 g/dL y predominio de polimorfonucleares, 89%) fue compatible con peritonitis bacteriana espontánea. El paciente evolucionó favorablemente tras tratamiento antibiótico.

A pesar de su excepcionalidad, resulta fundamental contemplar la peritonitis bacteriana espontánea en ascitis cardíaca dentro del diagnóstico diferencial para instaurar el tratamiento de forma temprana y reducir significativamente la morbimortalidad asociada.

## Abstract (en)

Cardiac ascites accounts for approximately 5% of ascites cases and is typically characterized by high protein concentration, which is thought to confer protection against spontaneous bacterial peritonitis. Nevertheless, spontaneous bacterial peritonitis may develop in the setting of advanced heart failure due to intestinal congestion and impaired perfusion.

We present the case of a 65-year-old male with advanced heart failure, a left ventricular ejection fraction of 19%, severe tricuspid regurgitation, and pulmonary hypertension, who presented with dyspnoea and progressive abdominal distension. Imaging excluded portal hypertension and cirrhosis. Ascitic fluid analysis revealed a protein concentration of 3.5 g/dL, a serum-ascites albumin gradient of 2.2 g/dL, and 89% polymorphonuclear cells, confirming spontaneous bacterial peritonitis. The patient responded favorably to antibiotic therapy.

Although uncommon, spontaneous bacterial peritonitis should be considered in cardiac ascites, as prompt recognition and treatment are crucial to reduce morbidity and mortality.

## INTRODUCCIÓN

La ascitis se define como la acumulación patológica de líquido libre en la cavidad peritoneal, resultado de un desequilibrio entre los mecanismos de formación y reabsorción del mismo^1^^,^^2^. Constituye una manifestación clínica de diversas enfermedades sistémicas o locales como cirrosis hepática asociada a hipertensión (la más frecuente), neoplasias, insuficiencia cardiaca, enfermedad renal o procesos infecciosos^1^^,^^3^. La ascitis de origen cardiaco representa aproximadamente el 5% de los casos, con diferencias fisiopatológicas y clínicas respecto a otro origen^1^^,^^4^.

Se presenta el caso de un varón con antecedentes de cardiopatía isquémica y disfunción ventricular que presentaba ascitis de origen cardíaco con cultivo negativo. El diagnóstico de peritonitis bacteriana espontánea permitió el tratamiento con ceftriaxona y el manejo del episodio de descompensación cardiaca aguda, logrando una evolución favorable.

## CASO CLÍNICO

Paciente varón de 65 años, que acudió a urgencias por aumento progresivo del perímetro abdominal, iniciado una semana previa a la consulta, acompañado de disnea grado III según la clasificación de la *New York Heart Association* y ortopnea de dos almohadas. Tenía antecedentes de cardiopatía isquémica, fracción de eyección del ventrículo izquierdo severamente reducida (19%), dilatación severa del ventrículo derecho, insuficiencia mitral moderada, insuficiencia tricúspidea severa y signos indirectos ecocardiográficos compatibles con hipertensión arterial pulmonar grave.

Negó fiebre, síntomas urinarios, vómitos, náuseas u otra sintomatología asociada; refirió cambio reciente de diurético. En la exploración física destacaba ascitis grado II y dolor a la palpación en hipocondrio derecho. A la auscultación pulmonar se observó abolición del murmullo vesicular en campos medios e inferiores del hemitórax izquierdo. 

**Figura 1:**
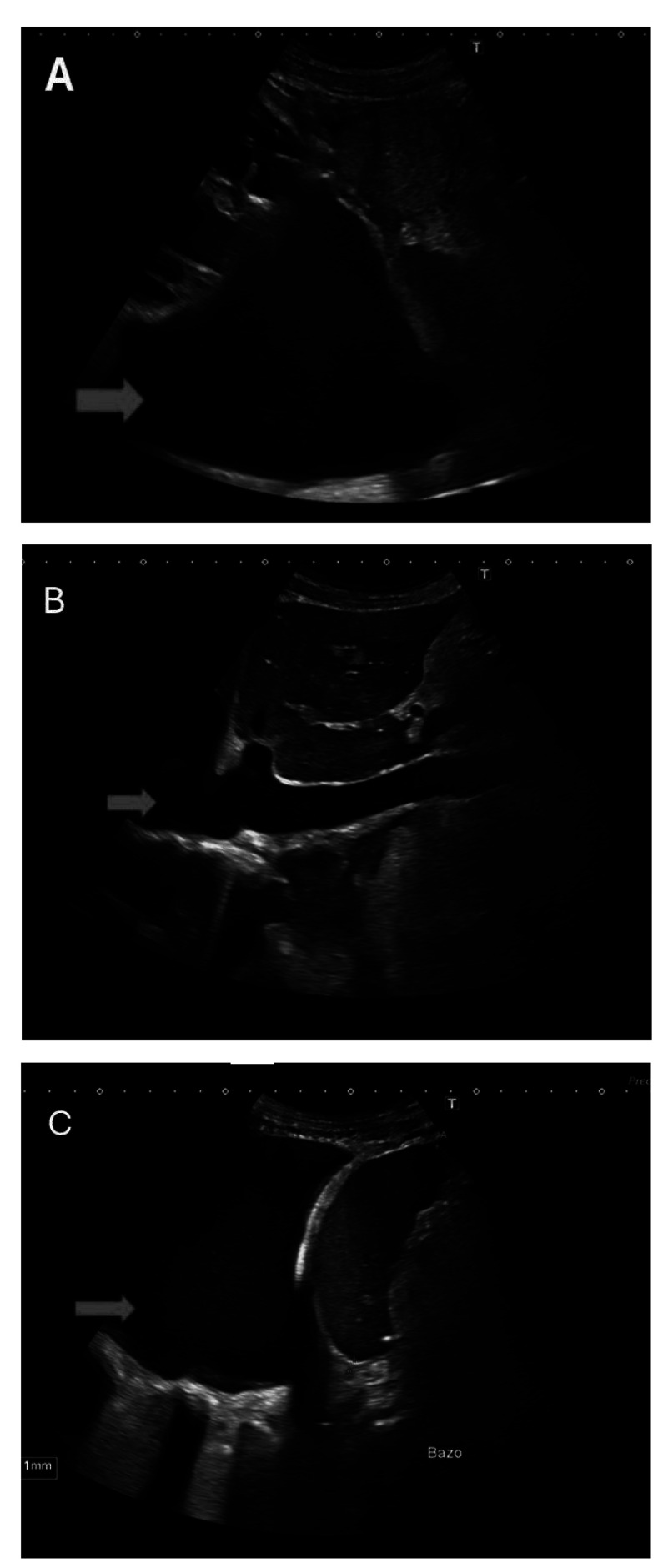
Ecografía abdominal. **A.** Plano coronal oblicuo (longitudinal) de hipocondrio derecho. **B.** Plano subcostal transversal hepático. **C.** Plano sagital oblicuo de hipocondrio izquierdo. Se observa la presencia de líquido ascítico (flechas rojas).

Se realizó analítica general que mostró elevación del péptido natriurético tipo B (NT-proBNP) con un valor de 21.900 pg/dL (valor normal, VN: 0-125). En la radiografía de tórax se observó derrame pleural izquierdo. La ecografía abdominal mostró un hígado de morfología y tamaño normales, con ecoestructura heterogénea, sin lesiones focales ocupantes de espacio. Las venas suprahepáticas presentaban calibre normal y flujo permeable en estudio Doppler. El eje esplenoportal era de calibre normal y con flujo conservado. Se objetivó ascitis de moderada cuantía (Figura 1).

Se realizó paracentesis diagnóstica con los siguientes resultados: 339 leucocitos/µL (89% polimorfonucleares, 11% mononucleares); proteínas totales: 3,5 g/dL (VN: 1.5-2,5); albúmina: 2,2 g/dL (VN: 1-1,5); gradiente albúmina sérica-ascitis (GASA): 2,2 g/dL, lactato deshidrogenasa (LDH): 108 U/l (VN: <200); glucosa: 88 mg/dL (VN: 50-100).

Ante la compatibilidad del líquido ascítico con peritonitis bacteriana espontánea (PBE), se inició tratamiento antibiótico empírico con ceftriaxona 2 gramos diarios durante 5 días, y se optimizó tratamiento diurético (se reintrodujo furosemida recientemente sustituida por torasemida, ante la sospecha de causa de descompensación ascítica, y se asoció espironolactona). Los resultados del cultivo de líquido ascítico fueron negativos. 

Tras una evolución favorable, fue dado de alta con posteriores revisiones en consultas externas, sin precisar nuevos ingresos hospitalarios por este motivo, ni presentar episodios de descompensación de insuficiencia cardíaca.

## DISCUSIÓN

La ascitis cardíaca es la acumulación de líquido en la cavidad peritoneal secundaria a insuficiencia cardíaca derecha descompensada o a condiciones que generan constricción u obstrucción del flujo cardíaco derecho^4^^,^^5^. La elevación de la presión en la aurícula derecha se transmite retrógradamente a través de la vena cava inferior y las venas hepáticas hacia los sinusoides hepáticos, provocando hipertensión sinusoidal y congestión hepática, que producen un exudado rico en proteínas^5^^,^^6^. La congestión venosa crónica también altera la perfusión hepática, generando isquemia, lesión biliar y fibrosis progresiva, lo que a su vez puede incrementar la presión portal y perpetuar la formación de ascitis. 

En casos de hipertensión arterial pulmonar, la ascitis cardíaca es rara y generalmente ocurre en etapas avanzadas^4^^,^^7^. Genera un aumento de la poscarga del ventrículo derecho, lo que provoca su dilatación y el desarrollo de insuficiencia tricúspidea funcional. Como consecuencia, se produce un incremento de la presión venosa sistémica^5^^,^^7^. Los pacientes con ascitis de origen cardiaco manifiestan síntomas de insuficiencia cardiaca (principalmente derecha, con edema periférico, reflejo hepatoyugular e ingurgitación yugular) que se acompaña de disnea, ortopnea y disnea paroxística nocturna^8^, algunos de los cuales estaban presentes en nuestro paciente.

La ascitis cardiaca, generalmente, presenta riesgo bajo de infección peritoneal debido a que el líquido ascítico es rico en proteínas, conservando su actividad opsónica y bactericida^3^. La ascitis hepática, por el contrario, suele tener menor contenido proteico y, por tanto, mayor susceptibilidad a la PBE, definida como la infección del líquido ascítico sin un foco intraabdominal evidente^1^^,^^9^. Para su diagnóstico, se debe realizar una paracentesis con análisis bioquímico, celular y microbiológico del líquido ascítico, donde los hallazgos más característicos incluyen: recuento de polimorfonucleares >250 células/µL, contenido proteico elevado (generalmente >2,5 g/dL en la ascitis cardíaca) y GASA >1,1 g/dL^1^^,^^3^. Estudios recientes sugieren, además, que los niveles séricos elevados de NT-pro-BNP pueden orientar hacia el diagnóstico de PBE en el contexto de insuficiencia cardíaca. Algunos de estos estudios obtuvieron una mediana para la ascitis por insuficiencia cardiaca de 6.100 pg/dL y han demostrado que la sensibilidad de valores superiores a 1.000 pg/dL para descartar cirrosis como causa de ascitis es casi del 100%^3^^,^^4^^,^^10^. Esto fortalece aún más el diagnóstico de nuestro paciente (con NT-proBNP = 21.900 pg/dL). En la Tabla 1 se recogen las principales diferencias entre las ascitis de origen cardíaco y cirrótico.

En nuestro caso, el paciente presentaba un líquido ascítico compatible con PBE, con GASA (2,2 g/dL) y proteínas totales (3,5 g/dL) elevados, sin cumplir los criterios de Runyon, que establecen una alta sospecha de peritonitis bacteriana secundaria cuando se cumplen al menos dos de estos tres criterios en el líquido ascítico: proteínas >1 g/dL, glucosa <50 mg/dL y LDH >225 U/L (nuestro paciente no cumplía los dos últimos). Esto, junto con la ausencia de cirrosis hepática, los antecedentes cardiológicos, los valores elevados de NT-proBNP y la evolución clínica favorable con antibioterapia dirigida a PBE, apoyó nuestra sospecha clínica de una PBE de origen cardiaco, a pesar del cultivo negativo. Esta discordancia es relativamente frecuente en los casos descritos en la literatura^11^^,^^12^ (Tabla 2).

**Tabla 1:** Principales diferencias entre ascitis de origen cardiaco y ascitis de origen hepático

Ascitis cardiaca	Ascitis hepática (cirrótica)
Etiologías principales	
- Insuficiencia cardiaca derecha	- Cirrosis hepática (alcohólica, viral, NASH)
- Pericarditis constrictiva	- Trombosis portal
- Enfermedad valvular derecha	- Budd-Chiari
- Hipertensión pulmonar avanzada	- Hipertensión portal no cirrótica
Mecanismo de producción	
Aumento de presión auricular derecha	Fibrosis y regeneración nodular
↓	↓
transmisión retrógrada a venas hepáticas	resistencia intrahepática
↓	↓
hipertensión sinusoidal	hipertensión portal
↓	↓
exudación de líquido rico en proteínas	aumento de presión hidrostática en sinusoides
↓	↓
drenaje linfático saturado	filtración de líquido hacia peritoneo; activación de SRAA y retención de sodio/agua
↓	
acumulación en peritoneo	
Mecanismo fisiopatológico clave	
- Congestión venosa pasiva crónica	- Hipertensión portal primaria
- Isquemia y fibrosis hepática secundaria	- Hipoalbuminemia
- Sobrecarga de volumen sistémica	- Vasodilatación esplácnica
	- Activación neurohormonal (SRAA, simpático, vasopresina)
Características clínicas	
- Ascitis menos abundante al inicio	- Ascitis progresiva
- Hepatomegalia	- Signos de enfermedad hepática crónica (ictericia, telangiectasias, arañas vasculares, encefalopatía)
- Edema periférico	- Edemas periféricos en estadios avanzados
- Signo de ingurgitación yugular	
- Ausencia de antecedentes de enfermedad hepática	
Características del líquido ascítico	
- Generalmente trasudado	- Inicialmente trasudado
- Proteínas relativamente altas (≥2,5 g/dL)	- Proteínas bajas (<2,5 g/dL)
- GASA ≥1,1 g/dL	- GASA ≥1,1 g/dL
- Células mononucleares bajas	- Densidad celular y glucosa baja
- Bajo riesgo de PBE	- Bajo recuento PMN en ausencia de infección
	- Riesgo elevado de PBE

NASH: esteatohepatitis no alcohólica; SRAA: sistema renina angiotensina aldosterona; GASA: gradiente albúmina sérica-ascitis; PBE: peritonitis bacteriana espontánea; PMN: polimorfonucleares.

La llamada *hipótesis intestinal* propone que la hipoperfusión y la congestión intestinal, características de la insuficiencia cardíaca congestiva, pueden conducir a alteraciones en la morfología intestinal, de la permeabilidad de la mucosa y del equilibrio de la microbiota, lo que facilita la translocación bacteriana y la liberación de endotoxinas^1^^-^^3^. Simultáneamente, se postula que la hepatopatía congestiva crónica puede inducir un daño intestinal crónico, predisponiendo también a la translocación bacteriana^6^^,^^9^. En nuestro paciente, la HTP, la disfunción ventricular y la insuficiencia tricúspidea severa, conducen a un estado de congestión hepática e intestinal que justificaría esta hipótesis como factor contribuyente al desarrollo de PBE.

Desde la presentación de los dos primeros casos de PBE en ascitis cardiaca en 1984^3^ y 1987^5^, se han publicado pocos casos hasta la actualidad (Tabla 2). En aquellos pacientes a los que se realizó ecocardiograma, la dilatación del ventrículo derecho, la insuficiencia tricúspidea y los signos indirectos de hipertensión pulmonar reflejan el proceso fisiopatológico subyacente descrito anteriormente (Tabla 1) y ayudan a explicar la aparición de la ascitis en este contexto. Así mismo, las características celulares y bioquímicas (alto contenido proteico) del líquido ascítico son compatibles con ascitis de origen cardiaco. En nuestro caso, se desconoce el factor precipitante de la descompensación, aunque sospechamos que se debió a un cambio en el diurético que empleaba hasta el momento.

**Tabla 2:** Principales características de los casos de peritonitis bacteriana espontánea en ascitis cardiaca descritos en la literatura

Estudio	Paciente	Líquido ascítico		Hallazgos
Autor	Sexo	Leucocitos (células/ µL)	ProBNP sérico	Ecocardiograma
Año	Edad (años)	PMN (% o células/ µL)	(pg/mL)	Prueba de imagen abdominal
País	Clínica cardinal	GASA (g/dL)		
		Proteínas (g/dL)		
		Cultivo		
Gómez del Olmo et al^2^	mujer	13.800	n/e	cavidades derechas dilatadas
2010	74	89%		IT severa
España	aumento abdominal, IY	-		PSP 61 mmHg
		-		No hepatomegalia
		*Lysteria monocitógenes*		
Canakis et al^3^	varón	1.115	3.915	PSP 67 mmHg.
2019	85	49%		sin hallazgos hepáticos
EEUU	letargia, confusión	1,9		
		3,3		
		-		
Zafar et al^1^	varón	-	n/e	n/e
2020	62	1.300		sin hallazgos hepáticos
EEUU	disnea, edemas y ascitis	4,3		
		4,3		
		-		
Patel et al^11^	mujer	23.000	4.497	VD dilatado
2019	52	92%		IT moderada
EEUU	dolor abdominal, ascitis, e IY	1,4		PSP 76 mmHg
		-		cirrosis
		*Candida glabrata*		
Ormachea et al^9^	varón	-	2.736	IT severa
2024	46	1.100		sin hallazgos hepáticos
EEUU	astenia y ascitis	1,4		
		>2,5		
		-		
Beckmann et al^5^	varón	-	n/e	VD dilatado
2024	75	663		IT severa
EEUU	disnea y desaturación	1,2		PSP 74 mmHg
		2,6		cirrosis con venas hepáticas permeables
		-		
Rossiter et al^12^	-	n/e*	n/e	n/e
2015	mediana edad	-		n/e
Canadá	ascitis	n/e		
		n/e		
		n/e		
Sandozi Mudassar et al^13^	varón	-	3.828	no
2023	71	520		cirrosis
EEUU	clínica gastrointestinal	2		
		4,1		
		-		
Karambelkar et al^14^	mujer	21.250	n/e	HTP (datos indirectos)
2020	72	-		IT severa
EEUU	distensión abdominal, vómitos, diarrea	1,2		cirrosis
		-		
		-		
Schutte et al^15^	varón	948	n/e	n/e
2022	37	-		n/e
-	disnea y anasarca	0,6		
		4,2		
		n/e		
Nuestro paciente	varón	339	21.900	dilatación VD
	65	89%		IT severa
	ascitis y disnea	2,2		datos de HTP
		3,5		sin hallazgos hepáticos
		negativo		

IY: ingurgitación yugular. HAP: hipertensión arterial pulmonar. PMN: polimorfonucleares. GASA: gradiente albúmina sérica-ascitis. PSP: presión sistólica pulmonar. IT: insuficiencia tricúspidea. VD: ventrículo derecho. HTP: hipertensión pulmonar; n/e: no especifica; -: dato no disponible; *: compatible con PBE.

En conclusión, el caso presentado subraya la necesidad de un alto índice de sospecha clínica de PBE en ascitis de origen cardíaco incluso con cultivo negativo, ya que se trata de una complicación inusual pero potencialmente grave si se retrasa el tratamiento de la insuficiencia cardíaca descompensada. La realización de paracentesis temprana y el análisis del líquido ascítico permitió el diagnóstico e iniciar el tratamiento adecuado, mejorando el pronóstico del paciente.

## Data Availability

Se encuentran disponibles bajo petición a la autora de correspondencia.
